# A service-learning experience in a free medical centre for undocumented migrants and homeless people

**DOI:** 10.1186/s13690-021-00530-6

**Published:** 2021-01-12

**Authors:** Giulia Civitelli, Marica Liddo, Irene Mutta, Bianca Maisano, Gianfranco Tarsitani, Maurizio Marceca, Gonzalo Castro Cedeno, Salvatore Geraci

**Affiliations:** 1 Department of Public Health and Infectious Diseases - Sapienza University of Rome, Piazzale Aldo Moro 5, 00185 Rome, Italy; 2 Caritas Medical Area, Via Marsala 103, 00185 Rome, Italy

**Keywords:** Global health education, Migrants, Refugees, Homeless people, University’s third mission

## Abstract

**Background:**

Service-learning experiences, informed by the realities of poverty and marginalization, are important for the education of future health professionals in order to commit them to tackling health inequalities and working with underserved populations. At the Caritas Medical Centre for undocumented migrants and homeless in Rome, students obtain an educational experience of service. The aim of this study is to try to measure the long-term impact of this experience on the professional and life choices of the student participants.

**Methods:**

A questionnaire was designed and distributed by email to all 19–29 years old participants in the experience. Responses were collected and analysed in a quantitative descriptive way and in a qualitative way using the knowledge, skills and attitudes model.

**Results:**

One hundred and seven students responded from the total 763 questionnaires distributed. Ninety-five percent of participants expressed a very high overall satisfaction, 93% declared that the experience influenced his/her future personal choices, and 84% found that the experience influenced their professional choices. Results were arranged into 6 categories of comments: knowledge about the realities of migration, poverty, and marginalization; relational skills; collaborative skills; attitudes towards migrants, poor people and others; Attitudes towards future professions; Attitudes towards life. A final category was listed with self-reflective questions related to the experience.

**Conclusion:**

This research shows the importance of service-learning experiences made during academic studies from young students of medicine and other faculties. Developing a relationship with marginalized and homeless people, within a voluntary service setting, can influence the future professional and personal choices of students. Universities should recognize the value of such experiences and establish partnerships with non-profit organizations to allow future health professionals to confront health inequities and commit themselves to their reduction.

## Background

It is essential for social and health professions students to gain a wide vision of health that is grounded in the theory of social determinants of health [1–3], as part of their education. The theory of social determinants of health is at the centre of a global health approach, which has spread in Italy through the Italian Network for Global Health Education (INGHE) [4, 5]. To reach transformative learning and contribute to educating agents of change agents [6], it is important not only to attend lectures on global health and related issues, but also take part in educative experiences especially in marginalized context. In Italy, such educational experiences are called “global health gyms” and they have a proven impact on the future professional and personal choices of students [7, 8].

Among the realities of marginalization, circumstances connected with undocumented migrants and homeless people are particularly appropriate to give to students alternative insights in health and health care. Medical education should face the challenges that come from those realities [9–11].

In Italy undocumented migrants can access the National Health System thanks to a special Straniero Temporaneamente Presente (STP) code for temporarily present foreigners. The code can be applied for at any time, without the person being unwell, is valid for 6 months and can be renewed. This system ensures equal access to all urgent and essential care for undocumented migrants. The code was established by law in 1995 and was subsequently upheld by law in 1998. Prior to these legal securities, undocumented migrants could only access health care services through emergency rooms, until voluntary associations organized free medical centres in the 1980s [12–15].

Caritas Medical Centre began activity in Rome in 1983. Caritas is a catholic organization committed to the assistance of poor and marginalized people [16], with a special educative and pedagogic mission for the whole society. The aim of Caritas is not to replace the government in taking care of the needs of the poor, but to start charity activities, which could foster community cohesion. Advocacy and commitment to rights are an important part of the work of Caritas, as well as education for the society. According to this vision, since the centre’s establishment, young university students could volunteer to engage with marginalized people in a medical centre with a low threshold of access. The possibility of voluntary student access is coherent with the vision of global health and represents a global health gym in the Italian system.

From 1985 to 2004 many young doctors carried out civil service at Caritas Medical Centre as conscientious objectors. Since 2001 Caritas Medical Area began collaborations with the Italian Secretary of Medical Students (SIMS), one of the four medical faculties present in Rome. Three times each year (in February, May, and October) ten places were available for students interested in knowing the reality of Caritas Medical Centre. Students were selected by SISM and, prior to starting the internship, they took an introductory course on migration and health where they learned the basis of socio-demographic aspects of migration, migrants’ health profiles, the relational and cultural aspects of migration, the law and politics related to migration and health, as well as insights into Caritas Medical Centre. At the beginning of the course, students usually answered an open questionnaire to share their expectations for the educative experience. Students attended the centre one afternoon every week, and they experienced the different aspects of the services: the welcome point, visits with general doctors, visits with specialists, work with nurses for wound dressing. At the end of the approximate four-month internship, students took part in an evaluation meeting.

Since 2011 medical students could also take part in another type of internship, called “Health on the road”. During the internship, they accompany social workers around the city and meet homeless people. They are encouraged to start a dialogue with the homeless, and understand if they can offer any assistance. The internship aims to help learners enter into a relationship with marginalized people as a fundamental starting point of a relationship of care.

Other young people and students attend Caritas Medical Centre as a volunteer, for the civil service or as interns from other faculties. The large majority of young volunteers were students of health professions faculties, mainly medicine and surgery.

After the internship students were engaged in the proposal, planning and participation of peer education activities at university in order to share their experiences with other colleagues. Peer to peer spread of these educational opportunities was fundamental in the past years.

## Methods

A questionnaire was designed to evaluate the medium and long-term impacts of a voluntary period spent at Caritas Medical Centre, for different types of internships or simply for voluntary work. There are few studies that investigate the long-term impact of educational experiences [7, 17, 18].

The questionnaire, developed under the direction of Caritas Medical Area after a basic literature review and especially with a participatory process among the organisers, was distributed online as a PDF file. The questionnaire included with closed and open questions, used the Likert scale for evaluation and was validated by submission to a small panel of “expert students”.

The questionnaire was sent by electronic mail to all students who gave their authorization for contact by mail. The beginning of the questionnaire asked participants to consent to participate in the research.

The questionnaire, composed of closed and open questions, investigates the characteristics of the responder, the actual study or work position, the type of internship, the overall satisfaction in taking part to the internship (using a 0–5 Likert scale), the influence on professional growth, on growth in the “social and relational dimension” and on professional, personal and spiritual choices.

Collected data have been analysed trough Excel (Microsoft Office version 16.0.11629.20246), using descriptive statistics. Variables have been expressed in absolute and percentage frequencies.

Qualitative data have been analysed using the knowledge, skills and attitudes model.

## Results

From 1983 to 2017 a total of 1310 young students, mainly from health professions (especially medicine) faculties, attended the Caritas Medical Centre. Among them, the questionnaire was sent to participants with an available mail address, in total 763. A total of 107 questionnaires were received (14% of the total) and a description of participants is described in Table 1.

**Table 1:** Description of participants

**Sex**		
F	70	66%
M	37	33%
**Age**		
21–25	11	10.3%
26–30	34	31.8%
31–35	31	29%
36–40	9	8.4%
41–50	8	7.5%
> =51	14	13.1%
**Type of experience**		
Medical student internship	60	56.1%
Civil Service/OC	21	19.6%
Voluntary service	23	21.5%
Other student internship	3	2.8%
	107	

Ninety-five percent of participants expressed a very high overall satisfaction (on a 6 point Likert scale from 0 to 5, 95% of participants selected 4 or 5, with a medium score 4.5). Eighty-two percent of respondents continued their commitment to migrants and poor people in various ways after the experience. Ninety-three percent declared that the experience influenced his/her future personal choices, and 84% found that the experience influenced their professional choices. Ninety percent of participants agree that the experience allowed him/her to enter in contact with the social problems of the city and with the life of poor and marginalized people. Eighty-five percent of participants think that this experience has improved his/her capacity to enter in a relationship with marginalized people.

The majority of people who took part to the survey affirm that they were able to discover the value of human dignity, improve their relational skills with poor people, discover a new trust in their profession, learn to withhold judgement and not be influenced by prejudices from other people, learn to collaborate, to welcome everyone and to deeply listen to the person who is in front of them.

Analysing the qualitative comments of students with the knowledge, skills and attitudes model, it was possible to describe those comments dividing them into 6 categories:

- Knowledge about the realities of migration, poverty, and marginalization
- Relational skills
- Collaborative skills
- Attitudes towards migrants and poor people (and in general towards every person)
- Attitudes towards future professions
- Attitudes towards life

We report some qualitative comments related with every category and some questions that emerged in students during the experience.

### Relational skills

- ✓ [This internship helps me to] Increase the ability of listening and comprehension of relational and emotional borderline situations.
- ✓ [This internship helps me to] Learn how to enter in relationship with fragile patients, even overcoming language barriers and creating a relationship of mutual trust.
- ✓ Every meeting and every relationship is an original, particular and unique moment…I learnt to take seriously every person that you meet regardless the role that you have.

### Collaborative skills

- ✓ Seeing a cohesive team of people with different specialization. Seeing how they persevere together. Seeing them always persist. Seeing them work gently and with passion. Seeing them also in their defects and difficulties and notice their more human side.
- ✓ I interacted with expert social workers and with people I met on the street who shared their experiences with me.
- ✓ From a personal point of view, I was impressed by the collaboration among professionals and volunteers.
- ✓ I learnt to look for people who have interests similar to yours and to give up other people who don’t understand and don’t appreciate you, because sharing the experience with other volunteers is something very precious and unique. I learnt to ask the help of others when I need (something not easy for me, as I was always used to working alone).

### Knowledge about the reality of migration, poverty and marginalization

- ✓ [To] See with my eyes what are the real needs of people, beyond books and academic texts. The reality and human relationships are the best teachers we could have.
- ✓ [Having] A greater comprehension of the condition of marginalization and of the subtended dynamics.
- ✓ Mainly, this experience allows me to meet global health, the social determinants of health approach and the vision of how those determinants influence the health and the life of people. I discover the dimension of advocacy and how it is important to act to promote and defend human’s rights.

### Attitudes towards migrant and poor people (and in general towards every person)

- ✓ I learnt to understand that beyond every human behaviour there is a hidden history and that even the most closed or resisting person can give a big lesson.
- ✓ The most notable teaching gives a greater and more conscious attention to the single person, taking each person exactly for who he is, without rushing or over structuring, at least at the beginning….I received a new look on the city and on its inhabitants, on the laws that rule it, on the limits that are everywhere, on the concept of equity (concerning health and other aspects) that is the necessary basis of every relationship.
- ✓ You learn to move the lens of the camera from yourself to the world that is around you and bring into focus that which you don’t want to see, that which you pretend to ignore in your everyday life because in many times it doesn’t mirror the criterion of decency that you learnt to respect. You understand what it means to be “normal”, how much luck there is in what we consider normal, how much richness is given from fate. The fragility of man isn’t sweet, touching, begging for help. More often it is furious, frustrating, disappointing, aggressive. It bites and attacks to defend itself; it consumes itself in humiliating realities. Life is a too strong an enemy to beat, it is able to strip a man of dignity.
- ✓ Thank to this experience I discovered my empathetic side that often cannot be shown in academic internships, both for a certain hierarchy and due to the lack of time. I learnt a lot on the relationship with the patient; it has to be founded on bilateral collaboration an not on pity, giving value to the person that is in front of you, giving him importance, tasks and dignity. It is fundamental to put yourself in the clothes of the other person to conduct a good anamnesis and to avoid medical errors, to understand the concrete needs and the everyday habits of this person. The objective is to adapt the best therapy for him (e.g., What does the patient eat? Where does he live? Does he have money to buy medicine? Does he understand all my suggestions or it is better to explain them in another language or in another way?). I understand that to do good things, it isn’t necessary to go in Africa, it is enough to go out from your home and look the sidewalk and the world with different eyes.
- ✓ I received a lot from those who have nothing material to offer. I challenge myself with the impotence to solve all the problems, together with the certainty that my presence, even if impotent, wasn’t indifferent to the patient. I realized that the little that each of us could do is very precious for others.
- ✓ I learnt that each of us has a past that maybe we cannot discover or understand at the first encounter, but its influence on the present cannot be ignored. This is valid even for a doctor or other health professional, who confronts fragile people and must suspect the presence of certain cultural/ familiar/ personal factors, and take them into consideration.
- ✓ I learnt to put myself in other’s shoes, to not take anything for granted, to get to the bottom of the situations, from life stories to the discover of places, without stop myself at the appearance. To take a minute more talking with the person who is in front of me, because the advantage that you receive is enormous and unexpected, both on a professional and personal level (initially, this was some something that was not easy for a shy person like myself). I learnt to turn away in the face of uncomfortable situations of marginality and disadvantage, because with the rights means it is possible to move close to these realities and offer concrete solutions.
- ✓ I learnt to understand the importance of the context of origin of the other person, the past life and the actual life conditions. For migrant people, for example, I learnt to understand that there was a life before their arrival in Italy, and what led to the situation in which we met. I learnt to knock over prejudices. I learnt to recognise differences in the ways of thinking and of facing life that could be influenced from culture, religion or life.
- ✓ The meeting with other people, put yourself at the same height, or maybe in a lower level (which is absolutely not an action of submission, but a chance to look at the person who is in front of you with another point of view.
- ✓ I learnt to not judge others only from the appearances. I learnt how much is important to listen. I learnt that smiles are a universal language.
- ✓ I learnt to enter in relationship with frail patients even overcoming language barriers and creating a relationship of reciprocal trust.

### Attitudes towards the future profession

- ✓ I’ve always thought that be a doctor is a mission. After this experience I understand that I want to work with people in the margins. To help them to return in the community from which they are excluded in their free time, to take care of them where the means are not sufficient for the work of doctors.
- ✓ I understand that I want to be a doctor who is able to act in every moment and context, to the best of my abilities, with every available means, without giving attention to the limits imposed by a specialization, by professional hierarchy, by ambition, etc., but devoting myself with body and soul to the patient and to the responsibilities for him. Frequenting the Caritas clinic helped me to tackle moments of doubts and uncertainty during the study, because I realized the final result of a knowledge that, once applied, is a continuous incentive and motivation.
- ✓ This experience has certainly influenced the type of doctor I would like to become.
- ✓ For me the experience in Caritas has been a real break. I understood that my approach to health has always been across-the-board and I had more clear idea of my professional future. I have chosen public health and global health.
- ✓ I’ve chosen to dedicate lot of my time on a research to migration and health to try to give scientific dignity to the needs of the patients of the clinic. Even those in a condition of poverty deserve the best possible therapy, supported by the best scientific evidence.
- ✓ Both the experience in the clinic and with the project “Health on the road” confirmed my interest in people who live at the margin of society.
- ✓ I’ve understood the type of doctor I would like to become: a doctor who is able to take care of people, giving them attention and love.
- ✓ Choose a career committed to poor people.
- ✓ I realize that there is a medicine dedicated to help the neighbour and which considers the person the centre of the work, diagnosis and therapy.

### Attitudes towards life

- ✓ I learnt to overcome lots of difficulties, which seemed insurmountable, but which scaled down in front of the pain of the others.
- ✓ For sure, I’ve participated to this experience to put myself to the test, to improve myself and to know myself better, and this is what has happened.
- ✓ I realized that a lot of things in the world where we live are not working and I’ve understood that I don’t agree with that situation, and this influences my life choices.
- ✓ Welcome the other person, when he or she is frail, sometimes demanding, bring me to put in discussion my faith highlighting the difficulty to welcome the other person for who he or she is.
- ✓ Meeting not only with migrants, but also even with people (volunteer and workers) who are dedicating all their life to this mission impressed me a lot. Thank to experiences like that, I could say: it is possible to live a life like that, in spite of the different signs that could come from the undergraduate and postgraduate academic context.
- ✓ I bring this experience in my heart and I’ll never forget that this allowed me to understand where to invest my time and my future energies, and helped me to come out of myself and to not feel ashamed of myself

### Questions

The internship in Caritas medical centre also raised questions on social responsibility, on the reason for injustices, and on which kind of interventions should be carried out at institutional level.

- ✓ The biggest question was a recurrent “why”, which was oriented to the question of fate: Why are there enormous differences of difficulties that two men have to face? Why are there violated childhoods? How and to what extent does context influence the behaviour, the mentality of every one of us, making individual, legal and professional justice impossible?
- ✓ Meeting people in conditions of marginalization has brought forth a lot of questions in me. Which kind of doctor do I want to be? What does service really mean and why do I want to experience it? To absolve myself or to meet the other person in a gratuitousness? Am I really able to go out from myself and to enter in relationship with others?
- ✓ I ask myself why we allow that there is such inequity with the distribution of richness, without doing anything to reduce these differences.
- ✓ What really matters in life, despite my objective wellbeing, I am often not able to smile with the heart like people in need can do sometimes? What really matters for a patient: medicine, diagnosis, or the smile, help and welcome of a doctor? Do I want to serve my ambition, the expectations of others or shall I feel free to receive life in giving my time, my skills to who is in poverty and marginalization? Which kind of doctor do I want to become?
- ✓ Do I listen enough to the patients?
- ✓ What can I do, here and now, and in the future?
- ✓ Which are the real priorities of life?

## Discussion

There is evidence and debates in the scientific literature related to the hidden curriculum and its effect on choices of future health professionals [19–21]. The expression “hidden curriculum” refers to the “processes, pressures and constraints which fall outside… the formal curriculum, and which are often unarticulated or unexplored [22].” The negative effects of the hidden curriculum on the intention of medical students to work with the underserved have already been demonstrated.

The internship in Caritas Medical Centre could be an example of community-based education and service-learning experience. Service learning, rooted in the pedagogical theories of John Dewey and Paolo Freire, is characterised by a combination of active community participation and ongoing reflection. Ideally, service learning helps medical students rediscover their initial, altruistic reasons for studying medicine. The main components of service-learning overlap with the principles of community-based participatory research, and are used to increase community engagement. Because of the overlapping goals of service learning and community-based participatory research, it is not surprising that service-learning projects are often described as “community-based education” [23]. There is evidence that community-based education motivates and prepares students to work in community health care, especially in realities of marginality and exclusion [24–26]. Service learning provides important theoretical and practical frameworks for local/global health education, whose aim is to tackle and reduce health disparities both domestically and internationally [27].

The survey responses among the participants to the internship or to the volunteer period in Caritas Medical Centre showed that this experience was not neutral for the majority of young people. In particular, the qualitative results demonstrate the importance of this kind of experience. Self-reflection and reflective exercises should become an integrating part of the internship.

The students met, in the majority of cases for the first time, a primary health care service for undocumented migrants and homeless people. The points of strength of the experience are connected, first of all, with the people they meet: poor people that are usually excluded from or discriminated within the public health services. Secondly, students could collaborate and learn from volunteers, who are motivated to spend their time with marginalized people. Volunteers became inspiring teachers of life lessons, and the relationship with them could be important for their future choices.

The authors believe that it is important to introduce students to the experience with a course on “migration and health” and on the reality and approach of Caritas services. At the beginning of the course it is important to collect student’s expectations on the experience. During the internship is also important to find some informal moments to listen to student’s feedback and impressions and to accompany them on the experience. At the end, an evaluation meeting is a good occasion to speak about the problems and point of strengths of the experience.

The authors are aware that a limit of this study is the answer rate among participants. This could be explained in some cases with the time distance between the internship and the mailing of the questionnaire.

Another limitation could be related with the fact that the majority of the authors are directly associated with this study, as they work or has worked in Caritas Medical Centre. This could be also a point of strength, because the authors are able to report evidence and suggestions from their everyday presence in Caritas Medical Centre. Knowing the reality of universities, especially of medical faculties, authors believe that it is fundamental to propose to students the possibility to do experiences outside the academic rooms and outside the university’s hospitals. Especially, it is important to give the possibility to enter in contact with the reality of migration, marginalization and poverty.

The real objective of the internship is to give a contribution to the education of future health professionals, to prepare them to defend and promote the right to health of every person, without any exclusion. As the educational activities of Caritas Health Area continue from more than 20 years, it is now possible to affirm that people who made an experience there are more prepared and sensitive in the work with migrants and poor people. They could make a difference in public health services, as the authors are experiencing, especially in this period of International outbreak of novel SARS-CoV-2 coronavirus infection. It is no coincidence that a past internship in this field is considered a good point for the selection of people.

## Conclusions

This research shows the importance of service-learning experiences made during academic studies from young students of medicine and other faculties. The relationship with marginalized and homeless people, which take place in a voluntary service where the person is the centre of the work, could influence the future professional and personal choices of students. This experience helps to develop relational and collaborative skills, knowledges about the reality of migration and poverty and attitudes towards migrants and poor, the future profession and life. The internship should be accompanied by a self-reflection exercise and dialogues with workers or expert volunteers. Unfortunately, Italian universities usually don’t consider this kind of experiences as a part of the curriculum, even if there is evidence of their importance in the scientific literature. The authors believe that academic courses should establish a partnership with no profit organizations directly committed in to understanding the realities of poverty and inequity [28]. The participation of students in these experiences should be voluntary and the academic staff and the staff of no-profit organizations should jointly supervise the experiences of the students. Service-learning experiences could play an important role in preparing future health professionals who are committed to tackling health inequities and acting for social justice.

## Data Availability

The datasets used and/or analysed during the current study are available from the corresponding author upon reasonable request.

## Abbreviations

GH: Global health
SISM: Italian Secretary of Medical Students
STP: Temporarily present foreigners
